# Immune cell dynamics and mechanisms of epithelial injury in celiac disease

**DOI:** 10.3389/fimmu.2026.1766513

**Published:** 2026-01-29

**Authors:** Irene Marafini, Silvia Salvatori, Edoardo Troncone, Pasquale De Vico, Elena De Cristofaro, Giovanni Monteleone

**Affiliations:** 1 Department of Systems Medicine, University of Rome “Tor Vergata”, Rome, Italy; 2 Gastroenterology Unit, Fondazione Policlinico “Tor Vergata”, Rome, Italy; 3 Department of Anesthesia, University of Rome “Tor Vergata”, Rome, Italy

**Keywords:** CD, gliadin, HLA-DQ2/DQ8, T cells, transglutaminase 2

## Abstract

Despite continuous exposure to dietary and microbial antigens, the intestinal mucosa maintains a delicate balance between immune activation and tolerance. This equilibrium depends on the integrity and regulatory functions of the intestinal epithelium and associated immune cells. In the case of celiac disease (CD), gluten ingestion disturbs this equilibrium in people with a genetic predisposition (those with HLA-DQ2 or HLA-DQ8 alleles), and this results in chronic inflammation and villous atrophy. Tissue transglutaminase 2 (TG2) modifies gluten peptides, thus enhancing their affinity for HLA-DQ2/8, with the downstream effect of triggering CD4^+^ T cell–mediated Th1 responses dominated by IFN-γ and IL-21. The same cytokines along with IL-15, which is released by the epithelial and dendritic cells, stimulate the activation of cytotoxic intraepithelial lymphocytes that, in turn, kill enterocytes. Additional innate pathways, including those induced by gliadin-derived peptides, α-amylase/trypsin inhibitors, and type I interferons, further amplify epithelial stress and immune activation. Crosstalk between immune and stromal cells and defects in counterregulatory mechanisms contribute to persistent tissue injury. Emerging evidence implicates the gut microbiota in modulating both gluten-dependent and -independent immune responses through protease activity and barrier regulation. We here review the available evidence supporting the role of immune cells in CD-associated tissue damage and discuss the basic mechanisms by which this destructive immune response is amplified.

## Introduction

Under normal conditions, the gut contains a significantly higher number of immune cells than other tissues. This state of minimal inflammation, which derives from the continuous stimulation of the intestinal immune system by dietary and microbial antigens and does not affect the digestive or absorptive functions, is tightly regulated by numerous mechanisms (1–3). The intestinal epithelium, a principal interface between the host and the environment (4), consists of different types of cells, such as absorptive enterocytes, goblet cells, Paneth cells, tuft and cup cells, microfold (M) cells, and enteroendocrine cells (5). All epithelial cells are derived from proliferative progenitors in the crypts of Lieberkühn at the base of the villi. Tight junctions uphold epithelial integrity, while goblet cell-derived mucus and Paneth cell-secreted antimicrobial peptides, such as defensins, lysozymes, C-type lectins, and cathelicidins, protect the epithelium from microbial attack through a MyD88-dependent mechanism (6).

Epithelial cells possess a large number of pattern recognition receptors (PRRs), among them Toll-like receptors (TLRs), NOD-like receptors, and retinoic acid–inducible gene-I–like receptors. Experiments on germ-free mice revealed that PRR expression is dependent on microbial colonization (7). As a result, the gut epithelium not only offers a physical but also a chemical barrier, which protects the host from allergens, toxins, microorganisms, and parasites that are naturally ingested with food (6).

**Figure 1:**
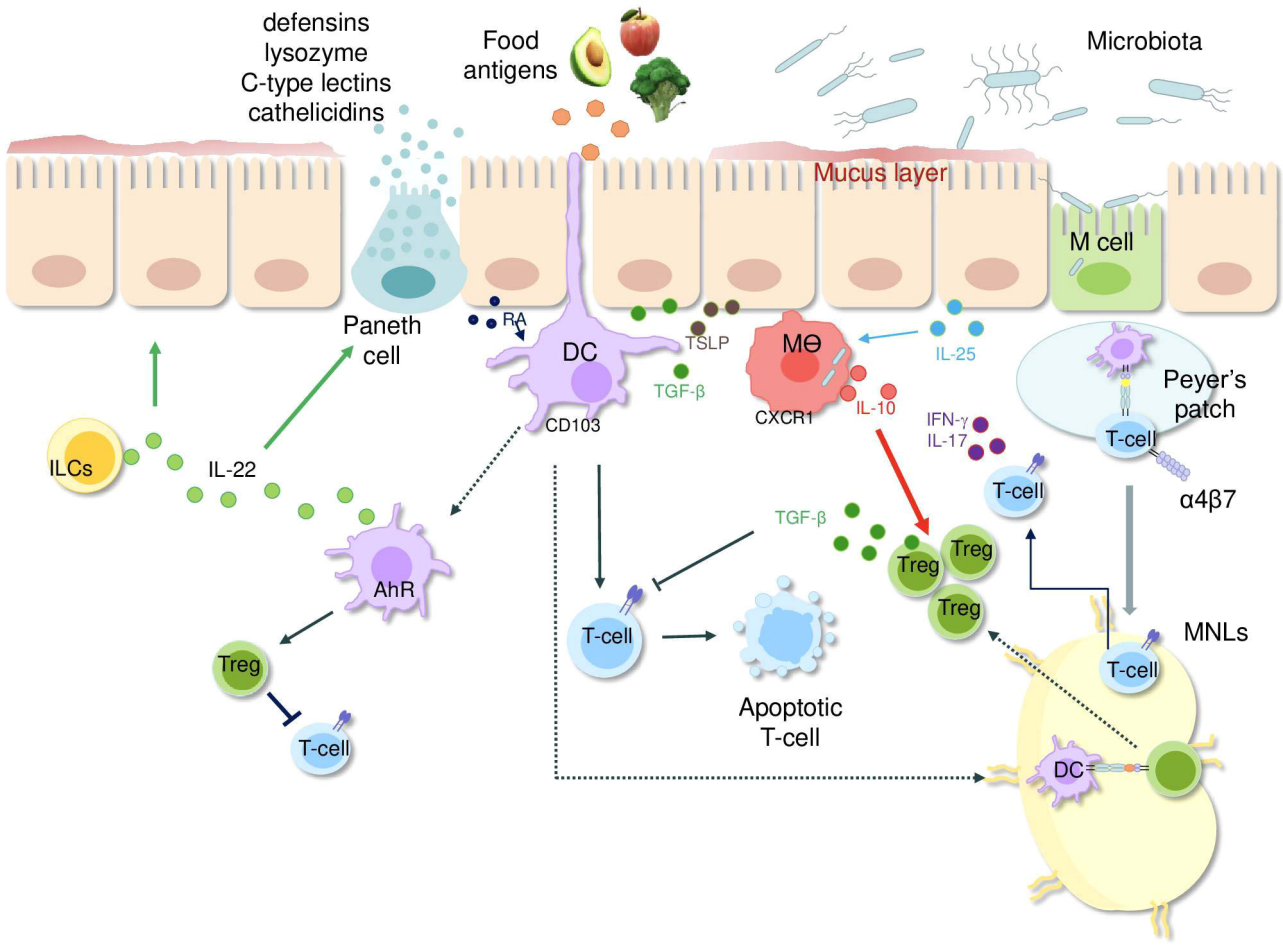
Simplified overview of key mechanisms maintaining intestinal immune balance. The epithelial layer, reinforced by tight junctions and antimicrobial peptides from Paneth cells (defensins, lysozyme, C-type lectins and cathelicidins), acts as a physical and chemical shield but still allows continuous sampling of luminal antigens (microbial and food antigens). These antigens reach Peyer’s patches via M cells or are captured directly by DC extending transepithelial projections and CX3CR1+ macrophages. After antigen uptake, DCs migrate to mesenteric lymph nodes and induce regulatory T-cell development through TGF-β and retinoic acid. Macrophage-derived IL-10 and TGF-β further sustain an anti-inflammatory milieu by limiting T-cell activation. Innate lymphoid cells support epithelial integrity and defensin production by releasing IL-22. Upon binding of dietary and microbial tryptophan metabolites to the aryl hydrocarbon receptor, DC promote the differentiation of regulatory T cells and secrete IL-22. Epithelial cells can also synthesize RA, TGF-β1, and TSLP, which promote tolerogenic DC activity, and IL-25 that supports the polarization of regulatory macrophages. Abbreviations: Ags, antigens; PRR, pattern recognition receptor; IL, interleukin; ILCs, innate lymphoid cells; DC, dendritic cell; Treg, regulatory T cell; M cells, microfold cells; RA, retinoic acid; IFN, interferon; MNLs, mesenteric lymph nodes; TGF, transforming growth factor; Mθ, macrophage; AhR, aryl hydrocarbon receptor; TSLP, thymic stromal lymphopoietin; RA, retinoic acid.

Despite this defense, luminal antigens can cross the epithelial barrier. For instance, in Peyer’s patches, M cells of the follicle-associated epithelium (FAE) take up antigens from the lumen and deliver them to the gut-associated lymphoid tissue (GALT) (5). Recent studies indicate that M cells might also play a role in antigen presentation via major histocompatibility complex (MHC) molecules. In organoid-based models, M cells expressing human leukocyte antigen (HLA)-DQ2.5 have been shown to present gluten antigens, implying a potential involvement of these cells in the development of (CD) (8). Apart from that, dendritic cells (DCs) take up luminal antigens through extending trans-epithelial protrusions, especially in the upper small intestine, without breaking the epithelial barrier (9) (**Figure 1**). The small intestine harbors two distinct subsets of DCs derived from common pre-DC precursors: intraepithelial DCs, which exhibit an immature-like phenotype and promote T cell hyporesponsiveness, and lamina propria CD103^++^CD11b^+^ DCs, which are mature and proinflammatory (10).

Additionally, macrophages, characterized by high CX3CR1 expression, capture luminal bacteria, fungi, and soluble antigens (11) (**Figure 1**). These cells possess strong anti-inflammatory potential given their ability to release interleukin-10 (IL-10), which supports regulatory T cell (Treg) proliferation and inhibits effector T-cell activation (11–13). After their activation, CD103^+^CD11b^+^ DCs travel to mesenteric lymph nodes and promote Treg differentiation via TGF-β1, retinoic acid (RA), and indoleamine 2,3-dioxygenase, the enzyme responsible for tryptophan metabolism (14, 15). The tolerogenic response is further supported by epithelial cell-derived thymic stromal lymphopoietin (TSLP) and IL-25, the latter being involved in the polarization of regulatory macrophages (**Figure 1**) (16–20).

Epithelial cells interact closely with innate lymphoid cells (ILCs) and intraepithelial αβ and γδ T lymphocytes (21–23). Certain ILC subsets produce IL-22, a cytokine essential for epithelial repair and Paneth cell differentiation (24, 25). Studies in human small intestinal organoid cultures have revealed that IL-22 does not directly regulate the regenerative capacity of crypt stem cells. Instead, it facilitates the formation of Paneth cells and enhances the expression of host defense genes, not only in Paneth cells but also in enterocytes, goblet cells, and tuft cells (26). IL-22 can also be produced by DCs in response to dietary and microbial ligand-driven activation of the aryl hydrocarbon receptor (AhR) (**Figure 1**) (27).

Activated T cells in a normal intestine express low levels of co-stimulatory molecules, have a limited capacity for proliferation, and are eliminated by apoptosis, thus preventing excessive immune activation (28–30). Disruption of epithelial integrity and/or impairment of regulatory pathways compromises immune tolerance to luminal antigens, leading to chronic intestinal inflammatory pathologies (1).

We here review the available evidence supporting the role of immune cells in celiac disease (CD)-associated tissue damage and discuss the basic mechanisms by which this destructive immune response is amplified.

## HLA-dependent gluten recognition and TG2-mediated immune activation in celiac disease

CD is an immune-mediated disorder affecting the small intestine after the intake of gluten, which is a protein complex found in wheat, barley, and rye. On a microscopic level, CD shows the features of increased intraepithelial lymphocytes (IELs), crypt hyperplasia, and different degrees of villous atrophy (31). These changes disrupt nutrient absorption, leading to a spectrum of clinical manifestations, from asymptomatic cases to severe malabsorption syndromes, and increasing the risk of complications (32–34). Patients with CD generate autoantibodies against deamidated gluten peptides and tissue transglutaminase 2 (TG2) (35, 36). These antibodies are not only the main features of the disease, but they are also indispensable instruments in the diagnosis and assessment of the disease condition. However, a definitive role for these antibodies in the pathogenesis of the disease has yet to be established.

CD develops in individuals carrying specific MHC class II HLA variants, highlighting the central role of genetics in disease susceptibility. The majority of patients (90–95%) carry the HLA-DQ2.5 haplotype (DQA105:01, DQB102:01), while a small proportion have HLA-DQ2.2 (DQA102:01, DQB102:02) or HLA-DQ8 (DQA103, DQB103: 02) (37–39). About 40% of people from the Western world have at least one of these alleles, but less than 2% of the carriers actually develop CD. This indicates that HLA variants are necessary but not sufficient for disease development, emphasizing the contribution of additional genetic, environmental, and immunological factors. Current evidence indicates that the HLA locus is responsible for about one-third of the genetic risk, while non-MHC susceptibility loci make up another ~15% of disease risk (40, 41). Non-HLA loci frequently refer to genes that regulate the immune system, maintain the intestinal barrier, and are involved in antigen presentation. A comprehensive sequencing and validation study recognized novel risk loci, showing strong associations with the disease in a targeted sequencing cohort and in large-scale UK Biobank analyses (42). Importantly, a number of the butyrophilin (BTN) 2A1 and BTN3A2 variants continued to be related to CD even after the HLA genotype adjustment, thus suggesting that the BTN-related pathways may proceed independently in disease predisposition.

The risk of CD is additionally dependent on the dosage of HLA genes. Those individuals who are homozygous for HLA-DQ2 or HLA-DQ8 are twice as likely as heterozygotes to develop the disease (43, 44). The reason for such an increased level of risk is the association of the highest surface expression of HLA-DQ molecules on antigen-presenting cells with the most efficient gluten peptide presentation (45). HLA-DQ2.5, in particular, is the most powerful one out of all the HLA variants, as it can bind a wide range of gluten peptides that are resistant to degradation in the gastrointestinal tract. On the other hand, HLA-DQ8 only binds a small number of peptides, which are more susceptible to enzymatic hydrolysis, whereas HLA-DQ2.2 binds only a few (41, 43, 46, 47). Moreover, HLA-DQ2.5 has been shown to have a higher peptide-binding affinity, hence allowing longer binding of gluten peptides to CD4^+^ T cells than HLA-DQ2.2 (48). One of the reasons for this functional difference is the polymorphism at DQα22, where the tyrosine residue in DQ2.5 can form hydrogen bonds with the peptide backbone, but the phenylalanine in DQ2.2 cannot (49). Taken together, all these data define a model where the magnitude and duration of gluten-specific CD4^+^ T cell responses determine whether CD develops or not.

One of the causes of gluten-related immunogenicity is the fact that the protein contains more proline than other compounds, and proline makes the protein resistant to gastrointestinal proteases. So partially digested gluten peptides accumulate in the gut lumen and become substrates for TG2-mediated deamidation. TG2 converts glutamine residues in gluten to glutamate, thus adding negatively charged residues, which increase the binding of DQ2 and DQ8, having positively charged pockets, to the negatively charged glutamate. This modification enables the production of HLA-DQ-gluten complexes on antigen-presenting cells, which, in turn, promote the very strong activation of gluten-specific CD4^+^ T cells (50–54).

Under normal conditions, TG2 is mostly inactive in the intestine. Nevertheless, inflammatory stimuli or cellular stress can increase the production and enzymatic activity of TG2, thus escalating immune responses to gluten (55, 56).

Apart from these molecular mechanisms, it is quite important to remember that protein defects of tight junctions and epithelial function may cause increased translocation of gluten peptides through the epithelium, thus making them more available to immune cells. Besides that, some microbial communities may affect not only gluten deamidation but also activation of TG2, hence suggesting that CD pathogenesis requires the interplay of genetic, environmental, and immunological factors (57, 58).

## Th1 and Th17 immune responses in celiac disease: roles of gluten-reactive T cells, cytokines, and regulatory pathways

The presentation of TG2-deamidated gluten peptides by antigen-presenting cells expressing HLA-DQ2 or HLA-DQ8 activates CD4^+^ T cells, thus inducing a Th1 immune response characterized by elevated IFN-γ and IL-21 production (52, 59). In line with this, intestinal mucosal cells from CD patients express STAT1, a transcription factor downstream of IFN-γ (60). Pharmacological inhibition of STAT1 in *ex vivo* organ cultures of CD mucosal explants blocks gliadin-induced upregulation of co-stimulatory molecules, including ICAM-1 and B7-2, which are involved in local immune activation. IFN-γ also promotes the expression of T-bet, a transcription factor critical for Th1 differentiation and function (61).

The transcriptional mechanisms driving Th1 differentiation in CD are not fully elucidated. Duodenal biopsies from gluten-exposed patients show high IL-18 expression, a cytokine that amplifies IFN-γ production (62). IFN-α, predominantly produced by antigen-presenting cells, is abundant in the CD mucosa and can promote T-cell responses to autoantigens (63). Cases of CD onset during IFN-α therapy for malignancies (64), as well as ex vivo studies using fetal gut explants, suggest that IFN-α can drive Th1 responses to gluten (65). Viral infections, which induce IFN-α, may contribute to the loss of oral tolerance in CD (66, 67). Notably, studies in mice have shown that gut-infecting viruses, such as Type-I Lang reovirus and murine norovirus, can disrupt intestinal homeostasis via IRF-1, leading to IL-12p40-producing DCs, gluten-specific IgG2c antibodies, TG2 activation, and delayed-type hypersensitivity to gluten (68, 69). In CD, CD103^+^ DCs use TG2 together with the LRP1 pathway to process gluten. This process enables DCs to deamidate and concentrate gluten peptides within lysosomes, thereby greatly enhancing their presentation to HLA-DQ2-restricted T cells (70).

The ongoing Th1-related immune response is further amplified by IL-15 (71). In human lamina propria lymphocytes, IL-15 activates Akt, thus enhancing IL-21 synthesis. Neutralizing IL-15 in mucosal cultures of CD patients reduces IL-21, while IL-21 blockade diminishes T-bet expression and IFN-γ secretion. Gluten stimulation of treated CD biopsies increases IL-21 expression, and IL-21 neutralization limits Th1 expansion. These findings are supported by studies in experimental models of CD. HLA-DQ8 transgenic mice overexpressing IL-15 in lamina propria and mesenteric lymph nodes exhibit altered DC activation, which promotes the expansion of IFN-γ-producing Th1 cells. In this model, RA and IL-15 activate the JAK-MAPK pathway in DCs, thus inducing IL-12p70 and IL-23 production (72).

CD mucosa contains elevated levels of Th17-type cytokines, and there is evidence that IL-21 contributes to amplifying such a production by gluten-non-reactive CD4^+^ and CD8^+^ T cells (73). Elevated IL-17A and IL-6 are also observed in refractory CD (RCD) type I (74). The CD-associated Th17 cell response may also depend on defective counter-regulatory mechanisms. In this context, Bowman et al. demonstrate that loss of A20’s ZF7 ubiquitin-binding domain drives pathogenic activation of Th17 cells, leading to increased IL-22 production and subsequent crypt hyperproliferation, induction of epithelial alarmins, and barrier dysfunction, thereby creating a microbe-driven inflammatory milieu reminiscent of small-intestinal pathology in CD (75). Importantly, CRISPR-mediated disruption of A20 in human CD4^+^ T cells recapitulates this IL-22-skewed phenotype, suggesting that A20 defects may serve as functional amplifiers of maladaptive T-cell responses in CD (75).

Effector cytokine profiles differ between active CD and RCD. IFN-γ and IL-21 are not elevated in RCD compared to controls, highlighting that Th1 induction in CD is gluten-dependent (74).

Supernatants from gluten-stimulated T cells trigger epithelial cell death, which is blocked by neutralizing IFN-γ antibodies. IFN-γ also upregulates HLA-E on epithelial cells, activating CD94/NKG2C-expressing cytotoxic IELs (76, 77), thus suggesting a role for IFN-γ in the development of villous atrophy. IFN-γ and IL-21 cooperate in generating anti-deamidated gluten peptide antibodies in mouse models with villous atrophy (77). Nevertheless, gluten-specific Th1 responses alone are insufficient to cause villous atrophy, because patients with potential CD, HLA-DQ2/DQ8 carriers with active adaptive immunity to gluten but lacking cytotoxic IEL activation, do not show villous damage (78, 79). Similarly, humanized HLA-DQ2/DQ8 mice with Th1 immunity to gluten, and IL-15-overexpressing transgenic mice with Th1 expansion, exhibit no villous atrophy (77).

Altogether the above data are consistent with the demonstration that the active phases of CD are characterized by an expansion of DC and monocyte subsets, which display a transcriptional profile aligned with their roles in antigen presentation and cytokine production (80). Recent large-scale single-cell transcriptomic analyses have further confirmed that the inflamed mucosa in CD patients is heavily infiltrated by monocytes and DCs exhibiting a proinflammatory phenotype, capable of promoting T-cell activation and exacerbating tissue inflammation (81).

Defective regulatory mechanisms further exacerbate immune activation. Active CD intraepithelial and lamina propria T cells show reduced AhR expression, which normally triggers suppressive signals (82, 83). Activation of AhR reduces inflammatory cytokines and cytotoxic effector molecules. In RCD, high Smad7 impairs TGF-β1 signaling, and antisense knockdown of Smad7 reduces inflammatory cytokine production (84). IL-15 also disrupts TGF-β-Smad3 transcriptional responses in CD T cells via c-Jun N-terminal kinase activation (85).

Altogether, these observations indicate that several effector cytokines and dysregulated regulatory pathways contribute to amplify both gluten-dependent and gluten-independent responses further exacerbating CD-associated mucosal inflammation.

## Innate immune dysregulation and epithelial stress responses in the pathogenesis of celiac disease

Multiple non-HLA genomic loci associated with CD encode proteins that regulate innate immune signaling, epithelial stress responses, and mucosal homeostasis (37). These findings reinforce the concept that dysregulated innate immunity is a fundamental determinant of disease pathogenesis. This paradigm is supported by observations in individuals with “potential CD,” who mount a gluten-specific adaptive immune response but do not initiate the characteristic epithelial stress program seen in active disease. As pointed out above, the absence of villous atrophy in these individuals indicates that adaptive immunity alone is insufficient to elicit mucosal destruction and that the full pathogenic cascade requires an innate immune component.

Gliadin peptides, beyond initiating adaptive T-cell responses, exhibit potent innate immunostimulatory properties. They activate monocytes, enhance DC maturation, alter DC-NK cell communication, and disrupt epithelial proliferative pathways, collectively contributing to the inflammatory milieu that culminates in villous atrophy (76, 77, 86, 87). Pionereing studies using ex vivo duodenal biopsy cultures showed that the α-gliadin peptide P31-43, which lacks T-cell stimulatory activity, induces robust innate immune activation (88). P31–43 peptide stimulates IL-15 production, increases expression of CD83, COX-2, and CD25 in CD3^−^ cell populations, and promotes enterocyte apoptosis (78). Importantly, pre-exposure to P31–43 primes the CD mucosa so that immunodominant gliadin epitopes can more effectively activate T cells through IL-15- and p38 MAPK-dependent mechanisms, indicating that subdominant peptides facilitate the development of antigen-specific adaptive immunity (89, 90).

At the mechanistic level, P31–43 interferes with the epithelial endocytic pathway due to partial sequence homology with hepatocyte growth factor-regulated substrate, a key regulator of endosomal maturation (79). This disruption prolongs the surface residence of receptor tyrosine kinases and sustains epithelial IL-15/IL-15Rα trans-presentation (89, 90). Prolonged IL-15 signaling promotes crypt hyperplasia, epithelial remodeling, and activation of cytotoxic innate lymphocytes. Furthermore, intestinal epithelial cells from CD patients express elevated levels of stress-inducible MHC class I-related molecules and HLA-E. *In vitro* exposure to gliadin digests or peptides such as P31–49 amplifies IL-15 production and upregulates these stress ligands (91). The pro-inflammatory effects of P31–43 require MyD88 and type I IFN signaling and are potentiated by poly I:C, implicating TLR3-mediated viral sensing pathways in the amplification of gliadin-induced inflammation (92).

Although gluten has long been considered the principal immune trigger in CD, additional wheat components, particularly α-amylase/trypsin inhibitors (ATIs), contribute substantially to innate immune activation. ATIs constitute potent ligands for the TLR4-MD2-CD14 complex and can directly activate monocytes, macrophages, and DCs (93). High concentrations of ATIs present in many processed and unprocessed wheat-containing foods can induce DC activation within mesenteric lymph nodes and promote intestinal inflammation. In genetically susceptible murine models, ATIs exacerbate gluten-induced immunopathology, whereas ATI-degrading bacterial strains, such as Lactobacillus, mitigate these effects (94).

A hallmark of untreated CD is the marked expansion of IELs within the intestinal epithelium (95). CD8^+^ αβ T-cell IELs in active disease acquire an NK-like phenotype, characterized by increased expression of activating receptors (NKG2C, NKG2D) and reduced expression of inhibitory receptors (CD94/NKG2A) (76, 96). These IELs exhibit potent cytotoxicity independent of gluten specificity and produce high levels of IFN-γ and granzyme B (76, 97, 98). Their cytolytic activity is mediated through interactions with HLA-E and other stress-induced ligands on enterocytes, reflecting a TCR-independent mechanism of epithelial injury.

Local cytokines within the mucosal environment play a pivotal role in IEL activation. IL-15 promotes CD8^+^ IEL expansion and upregulation of granzyme B and NKG2D, thereby enhancing cytotoxic function (99). IL-15 also antagonizes TGF-β1- and Treg-mediated immunosuppression, enabling sustained cytotoxicity (85, 100). IL-21 and IFN-γ synergize with IL-15, as demonstrated in transgenic mouse models that overexpress IL-15 in epithelial and lamina propria compartments and express HLA-DQ8; upon gluten exposure, these mice develop villous atrophy that can be mitigated by depletion of CD4^+^ or CD8^+^ T cells or neutralization of IFN-γ (77). Parallel findings in ovalbumin-based dietary antigen models confirm that epithelial IL-15 cooperates with antigen-specific CD4^+^ T cells to activate cytotoxic CD8^+^ IELs (87). IL-15 also enhances NKG2D-dependent killing of MICA-expressing enterocytes, and MICA expression is markedly increased in active CD via IL-15–dependent pathways triggered by gliadin and its derived peptides (91).

ILCs constitute another critical component of the mucosal innate immune system. These cells, which lack antigen-specific receptors yet depend on IL-7, are crucial mediators of tissue homeostasis, epithelial repair, and immune defense (101). Although ILCs represent approximately 10% of IELs (25), their rapid cytokine responses significantly influence mucosal inflammation. The major ILC subsets include NK cells, lymphoid tissue inducer cells, ILC1, ILC2, ILC3, and regulatory ILCs (24). NK cells form the dominant ILC population and exist as tissue-resident CD56^bright CD16^dim/– cells or circulating CD56^dim CD16^bright cells. ILC1s predominantly produce IFN-γ, ILC2s secrete type 2 cytokines, and ILC3s generate IL-17, IL-22, and TNF-α (102).

In active CD, NKp44^+^NKp46^+^ NK cells are significantly reduced in the epithelium, a pattern reminiscent of viral immune evasion and suggestive of a link between viral infections and CD susceptibility (103, 104). Conversely, NKp44-expressing ILC1s expand markedly in active and refractory CD, exhibit a pro-inflammatory phenotype characterized by secretion of IFN-γ and cytotoxic molecules, and correlate strongly with the severity of villous atrophy (105). Elevated numbers of TNF-α- and IFN-γ-producing ILCs, likely comprising ILC3s and helper ILC1s, are also observed in active disease (105). ILCs express TLR2, TLR3, and TLR9, enabling them to respond to microbial or viral mimetics such as poly I:C. In RAG1-deficient mice, systemic administration of poly I:C increases the frequency of TNF-α–producing ILCs and induces villous atrophy; depletion of ILCs abrogates this effect, highlighting a cytokine-dependent, lymphocyte receptor-independent pathway of epithelial damage.

Active CD mucosa is additionally characterized by increased apoptosis of epithelial and immune cells. Under physiological conditions, efficient clearance of apoptotic bodies by macrophages and DCs is essential to prevent secondary necrosis and to maintain tissue homeostasis. This process relies on phagocytic receptors such as CD36 and CD61 and on thrombospondin-1 (TSP-1), which mediates the bridging of apoptotic cells to phagocytes (106, 107). In CD, both the magnitude of apoptosis and defects in apoptotic cell clearance contribute to tissue injury. Active lesions display marked reductions in TSP-1, CD36, and CD61 expression and impaired phagocytic capacity (108). Elevated levels of IL-15, IL-21, and IFN-γ, cytokines central to CD immunopathogenesis, further suppress expression of these scavenger molecules, thereby promoting secondary necrosis and perpetuating inflammation.

## Bidirectional cross-talk between stromal and immune cells in the intestinal mucosa of celiac disease

Lamina propria stromal cells, including fibroblasts and myofibroblasts, are essential contributors to extracellular matrix synthesis and play a central role in maintaining intestinal stem cell niches (109). Beyond their structural functions, these stromal populations shape local immune responses through direct interactions with resident immune cells. Analogous to the supportive stromal networks found in the bone marrow, intestinal stromal cells can sustain long-lived plasma cells within the lamina propria. This survival support appears to depend on IL-6 and APRIL, together with chemokines involved in cellular retention and contact, such as CXCL12 (110).

**Figure 2:**
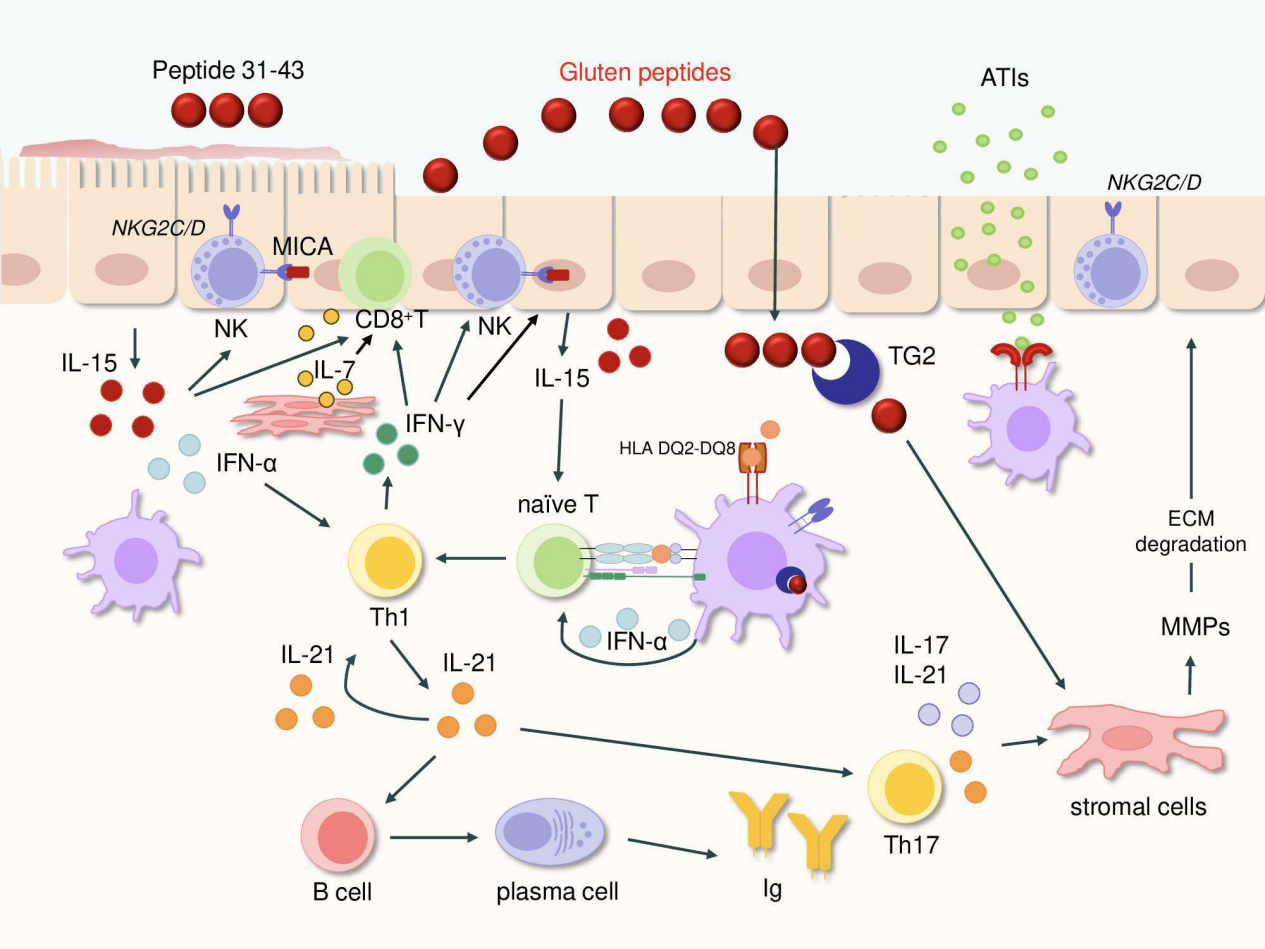
Figure summarizing the major pathogenic events leading to mucosal injury in celiac disease. Gluten peptides entering the lamina propria are deamidated by TG2 and presented by HLA-DQ2/DQ8–positive antigen-presenting cells, activating naïve T cells in the context of epithelial IL-15 and DC-derived IFN-α. This triggers a strong Th1 response with secretion of IFN-γ and IL-21: IFN-γ enhances cytotoxic CD8+ and NK-cell activity as well as contributes directly to epithelial damage, while IL-21 amplifies humoral and Th17 responses. IL-7 produced by stromal cells and epithelial cells further enhanced CD8^+^ IEL cytotoxicity. IL-17A and IL-21 stimulate stromal matrix metalloproteinases, contributing to extracellular matrix breakdown. IL-15 also increases the activation of NK receptors on intraepithelial lymphocytes, promoting killing of enterocytes expressing MICA. Non-gluten amylase/trypsin inhibitors activate innate immune TLRs, further escalating local inflammation. Abbreviations, IL, interleukin; IFN, interferon; DC, dendritic cell; TG2, transglutaminase 2; Ig, immunoglobulins; TNF, tumor necrosis factor; MMP, matrix metalloproteinases; HLA, human leukocyte antigen; BTN, butyrophilin.

Mesenchymal stromal cells (MSCs) also exert immunomodulatory effects. Ciccocioppo and colleagues reported that MSCs co-cultured with gliadin-specific T-cell lines inhibited T-cell proliferation and suppressed IFN-γ production (111). Moreover, MSCs altered T-cell subset composition, reducing CD4^+^ cells while expanding Foxp3^+^ Tregs. In contrast, studies by MacDonald and collaborators demonstrated that activation of Th1 cells in *ex vivo* fetal gut explants induces gut myofibroblasts to produce large quantities of matrix metalloproteinases, enzymes capable of degrading extracellular matrix components and promoting epithelial injury (**Figure 2**) (112, 113).

Richards et al. recently expanded this framework by showing that stromal-immune crosstalk is both spatially organized and transcriptionally rewired in active CD lesions. Using single-cell and spatial transcriptomics, the authors identified a distinct population of activated fibroblasts enriched at the villus–crypt junction, characterized by upregulation of CXCL9/10, OSM, IL-32, and BAFF. These stromal states were tightly linked to local expansion of cytotoxic CD8^+^ IELs and activated CD4^+^ T cells, suggesting that stromal “niches” generate chemotactic and cytokine gradients that orchestrate effector T-cell recruitment and retention (81). Further mechanistic insights into stromal-immune interactions indicate that myeloid-derived IL-1β and T cell-derived IFN-γ work together to shape pathogenic fibroblast states that support epithelial differentiation and remodeling in active CD.

Additional support for this notion comes from recent single-cell and spatial transcriptomic analyses by Fitzpatrick et al., who identified spatially organized stromal-immune interaction networks in CD. These networks link activated fibroblast states with cytotoxic and helper T-cell responses, as well as persistent epithelial remodeling, underscoring the crucial role of stromal-immune crosstalk in sustaining mucosal inflammation (114).

Recent experimental work using human organoids has provided direct mechanistic evidence that stromal cells actively shape gluten-driven immune activation in CD (115). In this system, the authors showed that gliadin stimulation rapidly induces IL-7 production predominantly within a lamina propria–like mesenchymal compartment, with minimal contribution from the epithelium. Notably, IL-7 was not merely a byproduct of inflammation; rather, it was both necessary and sufficient to drive CD8^+^ IEL cytotoxicity, acting upstream of NKG2C/D induction and fully recapitulating epithelial apoptosis even in the absence of gluten. Blocking IL-7 or NKG2C/D abrogated epithelial damage, supporting a model in which mesenchyme-derived IL-7 functions as a key amplifier linking CD4^+^ T-cell activation to cytotoxic IEL-mediated epithelial injury. These findings are consistent with biopsy data showing marked IL-7 overexpression in active CD compared with patients on a gluten-free diet. Together, these results position IL-7 as a central stromal-immune mediator of mucosal destruction and highlight the mesenchymal niche as a compelling therapeutic target. Spatial proteomics has further shown that IFN-γ acts directly on crypt epithelial cells to drive hyperplasia and profound metabolic reprogramming, indicating that additional factors other than stromal cell-derived molecules can control the abnormal epithelial responses in CD (116).

Collectively, these observations suggest that dynamic, bidirectional interactions between immune cells and stromal cells contribute significantly to the development of villous atrophy in CD.

## Heterogeneity of celiac disease

CD encompasses a broader biological spectrum than implied by the classical progression from gluten exposure to villous atrophy. Early phases of disease development can manifest as subtle epithelial stress responses, mild villous blunting, and selective increases in IELs, accompanied by a conserved epithelial metabolic program (117). These early molecular features, including type I IFN activity and activation of JAK/STAT-related transcriptional modules, precede overt architectural damage and help distinguish individuals who will later progress to mucosal enteropathy from those who remain in a potential, seropositive state (117). Beyond these prodromal signatures, the immune landscape varies substantially across patients: some individuals mount a restrained anti-gluten response characterized by preserved inhibitory NK-receptor signaling on IELs and a balanced IL-10/IFN-γ axis, whereas others develop a fully cytotoxic IEL phenotype associated with marked epithelial injury (118). Additional layers of heterogeneity emerge over time, as epithelial stress, immune activation, and stromal support pathways diverge to generate distinct disease trajectories, including gluten-dependent inflammation, gluten-independent immune activation, and progression toward aberrant IEL expansion and lymphomagenesis (119). These multidimensional variations across epithelial, immune, and stromal compartments underscore that CD is not a uniform entity but a continuum of related immunopathological states defined by distinct molecular programs and tissue responses.

## Conclusions

The findings presented in this article indicate that the development of gluten-specific CD4^+^ T-cell responses depends on coordinated interactions among gluten peptides, HLA-DQ2/8 molecules, and TG2. This adaptive response is essential for disease initiation but is not sufficient on its own to induce epithelial injury. Activation of cytotoxic CD8^+^ IELs represents a critical downstream event leading to epithelial destruction, and increasing evidence suggests that IL-15, together with cytokines produced by gluten-reactive Th1 cells, acts cooperatively to potentiate IEL cytotoxicity. Whether additional inflammatory mediators present in the diseased mucosa regulate this process remains unknown. Likewise, the precise intracellular pathways driving the expansion and activation of cytotoxic IELs are not fully defined, although indirect evidence supports the involvement of the JAK/STAT axis, given that both IL-15 and Th1 cytokines activate this pathway in T cells. Of note, malignant cells arising in enteropathy-associated T-cell lymphoma complicating RCD type 2 frequently harbor somatic mutations in JAK1 or STAT3 that confer a selective growth advantage. CD8^+^ IELs in CD also exhibit phenotypic similarities to CD8^+^ T cells bearing constitutive STAT3 gain-of-function mutations, including expression of NKG2D and genes encoding granzymes, perforin, and IFN-γ (120).

The mechanisms by which gluten peptides traverse the intestinal epithelial barrier remain incompletely understood. Current evidence supports a transcellular route involving endocytic trafficking (121). Similarly, the anatomical site at which TG2 encounters gluten peptides before engaging the immune system is not fully understood. Because TG2 requires extracellular Ca²^+^ for its enzymatic activity, the extracellular matrix beneath the epithelium is a plausible site for TG2-gluten interactions. Recent studies further suggest that TG2 may be released into the intestinal lumen during enterocyte shedding, where it could bind gluten peptides; TG2-gluten complexes may then be internalized by M cells in Peyer’s patches, leading to local antigen presentation and immune activation (122).

The contribution of B cells to pathogenic immunity in CD also remains incompletely defined. *In vitro* data indicate that B cells expressing a TG2-specific B-cell receptor and HLA-DQ2.5 can efficiently present deamidated gluten peptides to T cells, raising the possibility that anti-TG2 autoreactive B cells facilitate activation of gluten-specific CD4^+^ T cells and potentially contribute to epithelial damage (123). This hypothesis is reinforced by findings in HLA-DQ8 transgenic mice overexpressing IL-15 in the intestinal epithelium and lamina propria, in which villous atrophy induced by gluten and TG2 in a Th1-dependent manner can be prevented by B-cell depletion (124). Recent mechanistic insights have further clarified how and where TG2-reactive B cells become activated *in vivo*. Du Pré and colleagues demonstrated that small-intestinal lymphoid structures, particularly Peyer’s patches, serve as a critical site where TG2 and gluten converge to initiate the pathogenic B-T cell collaboration characteristic of CD (125). Using a highly controlled HLA-DQ2.5 murine model with adoptively transferred TG2-specific B cells and gluten-specific CD4^+^ T cells, the authors showed that luminal TG2 can translocate into the subepithelial dome of Peyer’s patches, where it is selectively internalized by TG2-specific B cells. Following oral administration of a TG2-gluten fusion antigen, these B cells underwent robust activation and expansion within Peyer’s patches and mesenteric lymph nodes, acquired the capacity to present gluten peptides to T cells, and differentiated into IgA-secreting plasmablasts. These findings provide *in vivo* evidence that gut-associated lymphoid tissue is not merely a passive sampling site but an active immunological niche in which TG2-gluten complexes are captured and processed. Therefore, therapeutic strategies targeting luminal TG2 availability or B-cell uptake could disrupt the early pathogenic checkpoint. CD19^+^ plasma cells constitute the predominant cell population in small intestinal biopsies from both uninflamed and active CD mucosa. These cells express surface MHC class II molecules, enabling them to recognize gluten peptides, present them to T cells, and clonally expand, thereby propagating the pathogenic immune response (126, 127). Notably, intestinal CD19^+^ plasma cells also express cytotoxic effector molecules capable of inducing epithelial cell death, suggesting an additional mechanism by which these cells may directly contribute to tissue damage in the gut (128, 129).

Alterations in the intestinal microbiota have also been documented in CD, although their causal relevance remains unresolved. Gliadin-reactive, HLA-DQ2.5–restricted T cells from CD patients cross-react with microbial proteases (130), and duodenal biopsies from active CD exhibit enhanced proteolytic activity against gluten substrates, correlating with an increased abundance of Proteobacteria, including Pseudomonas. Pseudomonas aeruginosa elastase can induce gluten-independent, PAR2.dependent inflammatory signaling: in HLA-DQ8 transgenic mice, the same protease synergizes with gluten to exacerbate inflammation and cause moderate villous injury (131). Active CD is further characterized by reduced duodenal expression of elafin, a human serine protease inhibitor. Supplementation of elafin via recombinant Lactococcus lactis ameliorates gliadin-driven immunopathology in the NOD/DQ8 mouse model (132, 133). Similarly, a serine protease inhibitor produced by Bifidobacterium longum NCC2705 attenuates gluten-induced pathology in the same model.

Regional epithelial expression of the transcription factor GATA4 regulates microbial colonization and immune homeostasis in the proximal small intestine by modulating retinol metabolism and luminal IgA. Mice lacking jejunal GATA4 exhibit dysbiosis and develop exaggerated Th17-mediated inflammation and barrier dysfunction upon Citrobacter rodentium infection (119). In CD, reduced GATA4 expression correlates with metabolic abnormalities, expansion of Actinobacillus, and heightened Th17 responses (134). Collectively, these findings demonstrate that microbial communities can modulate host immune pathways, potentially predisposing individuals to CD and amplifying gluten-dependent immunopathology.

From a therapeutic perspective, the challenges in CD go far beyond dietary management. While a strict gluten-free diet is still the foundation of treatment, a significant number of patients continue to experience persistent symptoms and/or villous atrophy, often due to ongoing gluten exposure or slow mucosal healing. A smaller subset of patients develops true refractory disease, which carries substantial morbidity and, in the case of type II, malignant potential (135). As a result, an expanding range of adjunctive pharmacologic strategies is being developed to complement the gluten-free diet. These include approaches aimed at reducing luminal gluten exposure (e.g., glutenases and gluten-sequestering agents), enhancing epithelial barrier function, and targeting key immune checkpoints in the pathogenic cascade (e.g., inhibiting TG2-mediated deamidation, blocking HLA-gluten presentation, and modulating cytokine pathways like IL-15-directed therapies). Clinical trial designs are increasingly incorporating histologic endpoints alongside patient-reported outcomes (136). In this regard, it is important to note that *in vitro* studies have demonstrated that the TG2 inhibitor ZED1227 prevents gluten-induced intestinal damage and inflammation. Additionally, it reduces gluten-induced duodenal mucosal injury in patients with CD, suggesting that TG2 plays a critical role as an adaptive element in the pathogenic cascade that leads to tissue damage (137, 138).

While numerous mouse models have been developed to study immune responses to gluten and to sever the roles of specific cytokines and cell subsets, none of them entirely recapitulates the genetic, immunological, and clinical features of human CD. It will be necessary to further improve CD-like models to unravel the order of pathogenic events and to carry out preclinical testing of novel therapeutic strategies.

Within this evolving landscape, refractory CD warrants special focus. Refractory CD type I is diagnosed by exclusion, following a thorough evaluation of dietary adherence and other potential causes of villous atrophy. In contrast, refractory CD type II is now recognized as a low-grade intraepithelial lymphoma, marked by abnormal or clonal IELs and a high risk of progression to enteropathy-associated T-cell lymphoma. This form of the disease necessitates management in specialized centers and underscores the need for more effective, mechanism-based therapies, going beyond conventional immunosuppression (139).
